# Evaluation of the Oesophagogastric Cancer-Associated Microbiome: A Systematic Review and Quality Assessment

**DOI:** 10.3390/cancers15102668

**Published:** 2023-05-09

**Authors:** Bhamini Vadhwana, Munir Tarazi, Piers R. Boshier, George B. Hanna

**Affiliations:** Department of Surgery and Cancer, Imperial College London, 7th floor Commonwealth building, Hammersmith Hospital, London W12 0HS, UK

**Keywords:** oesophageal cancer, gastric cancer, adenocarcinoma, squamous cell carcinoma, microbiome, diagnosis, metagenomics, biomarkers

## Abstract

**Simple Summary:**

Oesophagogastric cancer is the fifth most common cancer worldwide, with poor survival outcomes. Emerging data suggest that bacteria specific to cancer may play a role in earlier disease detection and treatment strategies. This study aimed to evaluate all microbiome studies identifying bacteria enriched in oesophagogastric cancer and appraisal of the methods. Eighty-nine studies demonstrated an enrichment of five bacteria in gastric cancer (*Lactobacillus*, *Streptococcus*, *Prevotella*, *Fusobacterium*, *Veillonella*) and three bacteria in oesophageal squamous cell carcinoma (*Streptococcus*, *Prevotella*, *Fusobacterium*). No differences were observed in oesophageal adenocarcinoma. Functional analysis supports the role of immune cells, localised inflammation and cancer-specific pathways in cancer progression. There is evidence that batch effects and contamination in sample analysis are poorly reported. The STORMS checklist provides a framework for high-quality microbiome studies. Whole-genome sequencing is recommended to provide key metabolic and functional pathway analysis of potentially important bacteria influencing cancer progression.

**Abstract:**

Objective. Oesophagogastric cancer is the fifth most common cancer worldwide, with poor survival outcomes. The role of bacteria in the pathogenesis of oesophagogastric cancer remains poorly understood. Design. A systematic search identified studies assessing the oesophagogastric cancer microbiome. The primary outcome was to identify bacterial enrichment specific to oesophagogastric cancer. Secondary outcomes included appraisal of the methodology, diagnostic performance of cancer bacteria and the relationship between oral and tissue microbiome. Results. A total of 9295 articles were identified, and 87 studies were selected for analysis. Five genera were enriched in gastric cancer: *Lactobacillus*, *Streptococcus*, *Prevotella*, *Fusobacterium* and *Veillonella*. No clear trends were observed in oesophageal adenocarcinoma. *Streptococcus*, *Prevotella* and *Fusobacterium* were abundant in oesophageal squamous cell carcinoma. Functional analysis supports the role of immune cells, localised inflammation and cancer-specific pathways mediating carcinogenesis. STORMS reporting assessment identified experimental deficiencies, considering batch effects and sources of contamination prevalent in low-biomass samples. Conclusions. Functional analysis of cancer pathways can infer tumorigenesis within the cancer–microbe–immune axis. There is evidence that study design, experimental protocols and analytical techniques could be improved to achieve more accurate and representative results. Whole-genome sequencing is recommended to identify key metabolic and functional capabilities of candidate bacteria biomarkers.

## 1. Introduction

Worldwide, oesophagogastric cancer is the fifth most common cancer, with over 1.5 million new cases and over 1.3 million associated deaths annually [1]. Prognosis remains poor despite advances in oncological and surgical treatment, which is primarily due to delayed diagnosis. Recent interest in the role of the tumour-associated microbiome in oesophagogastric cancer pathogenesis may offer an opportunity for new advancements in diagnostics, therapeutics and prognostication.

Previous studies have identified characteristic bacterial phenotypes related to intestinal cancers, including oesophageal, gastric and colorectal cancer [2,3]. Concurrent studies also found that the oral microbiome of gastric, colorectal, pancreatic and lung cancer patients was unique, offering a potentially non-invasive diagnostic window into the digestive tract [2,3,4,5]. In recent years, the emerging link between the gut microbiome and immunotherapy has provided a platform for potential treatment prediction. A less diverse gut microbiome following anti-PD-L1 immunotherapy plus chemotherapy in oesophageal squamous cell carcinomas may predict treatment responders [6]. Similarly, the gut microbiome has been shown to influence the therapeutic effect of anti-PD-L1 immunotherapy in colorectal cancer by modulating tumour-specific T-cells, and it plays a critical role in facilitating immunotherapy treatment in hepatocellular carcinoma, lung cancer and renal cell carcinoma [7,8,9,10]. In addition, microbial variance in pre-operative saliva samples predicted anastomotic leak and pneumonia following oesophagectomy [11,12].

Despite this interest, the microbiome of the upper gastrointestinal tract remains poorly understood compared to that of the colon and rectum [13,14]. This may be due in part to the presumed paucity of bacteria within the oesophagus and stomach and uncertainty as to their clinical significance. A notable exception is *Helicobacter pylori* and its established role in the pathogenesis of gastritis and gastric cancer [15]. Advances in sequencing techniques have led to a better understanding of microbial communities within the intestinal tract. Several disease processes within the gut have been shown to be associated with reduced microbial diversity and a change in bacterial composition [16,17,18]. It remains unclear whether this change is the cause or effect of a disease process.

## 2. Methods

### 2.1. Search Strategy

A systematic search was performed using the MEDLINE (OvidSP), EMBASE (OvidSP) and Cochrane databases to identify potentially relevant articles published between 1966 to 11 August 2022. A search strategy was formed with keywords and MeSH headings relating to “oesophageal cancer”, “gastric cancer” and “microbiome” used in combination with the Boolean operators AND and OR. Other databases, including the World Health Organisation International Clinical Trials Registry, ClinicalTrials.gov, ISRCTN Register and PROSPERO, were reviewed to identify ongoing studies.

### 2.2. Eligibility Assessment and Data Extraction

Studies that reported any aspect of the microbiome in patients with oesophagogastric cancer were selected from the electronic search results. Specific inclusion criteria were studies reporting named bacteria identified in patients with oesophageal and gastric cancer (any histological type) and compared to an appropriate control group of healthy subjects or patients with benign conditions of the upper gastrointestinal tract. All bio-sample types and methods for bacterial identification were included. Review articles, case reports, editorials, opinions, conference abstracts and articles not written in the English language were excluded. Two independent reviewers (BV, MT) screened all titles and abstracts to identify relevant articles for full-text review. A third reviewer (PRB) resolved any disagreements in article selection. Reference lists of all selected articles were also searched to identify additional relevant articles.

Data extraction from full-text review included: study design, cohort demographics, specimen type, microbiome assessment method, diversity characteristics (including α- and β diversity metrics) and bacteria identified at all taxonomic levels with *p*-values < 0.05.

### 2.3. Definitions of Groups

Subjects were classified into three groups: (i) patients with histologically proven oesophageal and gastric cancer; (ii) benign disease of the upper gastrointestinal tract, including superficial gastritis, atrophic gastritis; and (iii) healthy controls with no known pathology of the upper gastrointestinal tract. Premalignant conditions such as Barrett’s oesophagus, gastric intestinal metaplasia or any form of dysplasia were not included in the analysis.

### 2.4. Outcomes

The primary outcome was to identify bacterial enrichment specific to oesophagogastric cancer in comparison to a relevant control group. All bacteria identified were recorded, focusing on those reported in five or more studies. Other microorganisms such as fungi, viruses and protozoa were not recorded. Secondary outcomes included: investigating the relationship between the oral and cancer tissue microbiome; assessing the diagnostic performance of the cancer-associated microbiome; describing the microbial diversity and methodology appraisal.

### 2.5. Quality Assessment

The Newcastle-Ottawa Scale was used to assess the quality and risk of bias for case-control and cohort studies. Two independent researchers (MT, BV) graded the studies across three domains: selection of the study groups (adequacy of case definition, representativeness of the cases, selection of controls, definition of controls), comparability of the groups (based on design and accountability of confounding factors) and outcome of the studies (exposure, assessment methods, follow-up).

Studies were also evaluated using the Strengthening The Organisation and Reporting of Microbiome Studies (STORMS) checklist, recently developed for reporting on the quality of human microbiome studies [19]. It comprises a comprehensive structured 17-item checklist intended to standardise reporting of important quality and methodology parameters.

## 3. Results

### 3.1. Study Selection

**Figure 1:**
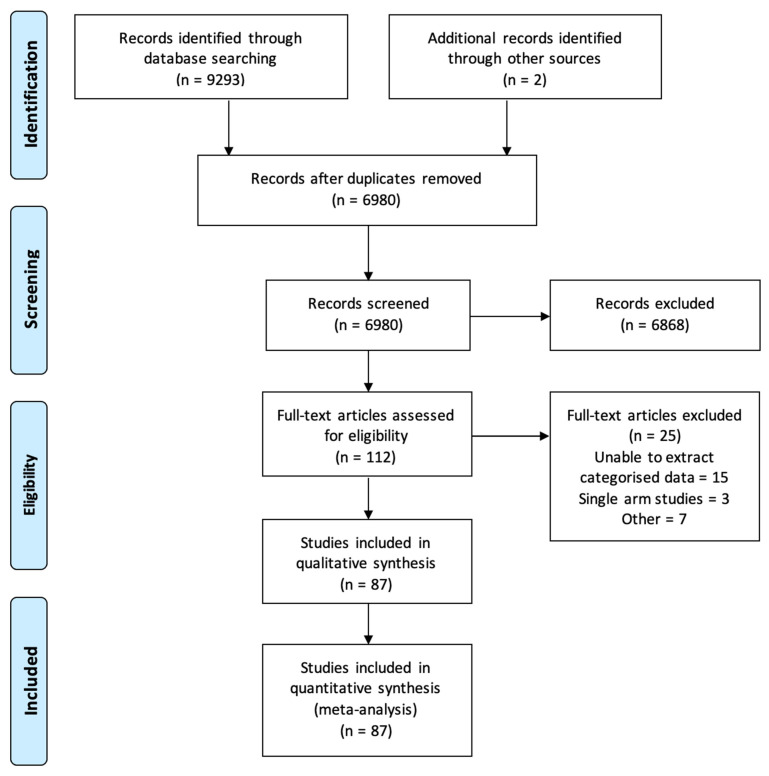
Flow chart demonstrating article selection.

A total of 9295 articles were identified from the online literature search. A total of 112 studies were selected for full-text review, of which 25 were excluded, as they were either a single-arm study (without control group), or they did not report relevant information (e.g., named bacteria). Final analysis included 87 studies: 56 studies assessing gastric adenocarcinoma [3,16,20,21,22,23,24,25,26,27,28,29,30,31,32,33,34,35,36,37,38,39,40,41,42,43,44,45,46,47,48] and 42 studies assessing oesophageal carcinoma (adenocarcinoma and/or squamous cell carcinoma) [12,35,38,49,50,51,52,53,54,55,56,57,58,59,60,61,62] (Figure 1). Two studies reported data from both oesophageal and gastric cancers [35,38].

### 3.2. Gastric Adenocarcinoma

Study characteristics: Fifty-six studies included analysis of 4545 patients with either: gastric adenocarcinoma (n = 4346); benign disease (n = 1364); or a healthy upper gastrointestinal tract (n = 2394) (Table 1; detailed study characteristics are provided in Table S1). The majority of studies were conducted in Asian populations (n = 46, 82%). Four studies were performed retrospectively from registered databases [22,63,64,65]. The majority of studies were prospective case-control studies, where the control group comprised either unrelated healthy (non-cancer) patients or healthy tissues samples (adjacent to the tumour) acquired from cancer patients.

Sample type: Microbial assessment was performed in various biological matrices, including upper gastrointestinal tract tissue (n = 34, 61%); faeces (n = 11, 19%); oral samples (saliva, n = 6, 10%; tongue coating, n = 3, 5%; plaque, n = 2, 3%); gastric/oesophageal fluid (n = 5, 9%); serum (n = 2, 3%); and urine (n = 1, 2%).

Method for bacterial identification: Significant heterogeneity was observed in the sequencing technique used to examine the microbiome. The majority of studies (n = 48, 86%) used 16S rRNA gene sequencing predominantly targeting the V3-4 hypervariable region. Four studies that utilised 16S rRNA gene sequencing did not specify which variable region was targeted [22,23,29,37,46]. Two studies employed primary bacterial culture from acquired tissue samples with subsequent 16S rRNA V3-4 sequencing [12,20], and two studies utilised shotgun sequencing and terminal restriction fragment length polymorphism [21,31]. Public datasets including The Cancer Genome Atlas and Memorial Sloan Kettering Cancer Center have been used to pool samples for big dataset analysis.

**Figure 2:**
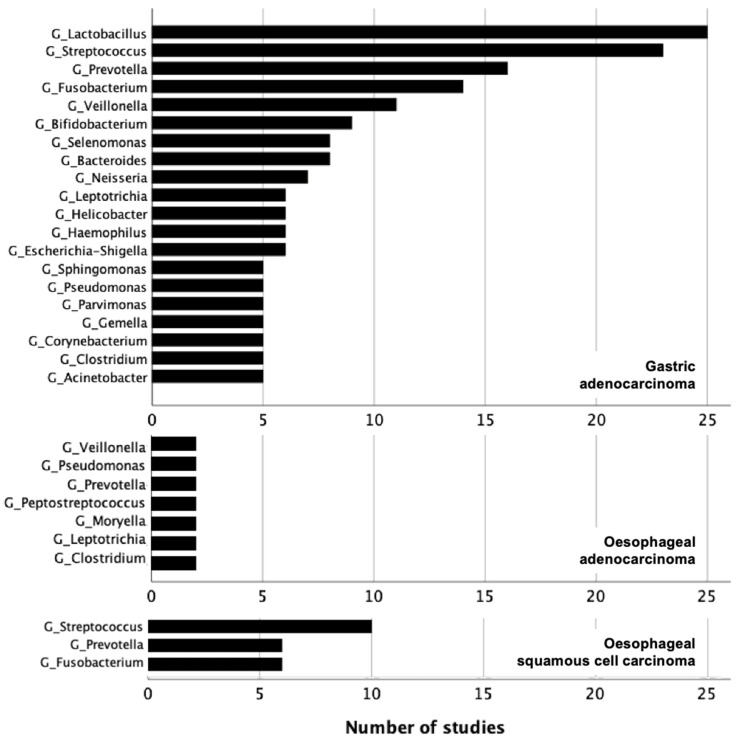
Bacteria enriched in oesophagogastric carcinoma across all included 89 studies. G = genus.

Bacteria enriched in gastric adenocarcinoma: Taxonomic classifications to the genus level were reported by the majority of studies. A total of 352 bacteria were enriched in gastric cancer at the following levels: 18 phyla, 15 classes, 31 orders, 50 families, 149 genera, 87 species and 2 strains. Bacteria that were reported by ten or more studies (36/357, including all taxonomic levels) are shown in Figure 2. Full details of bacteria identified in association with gastric adenocarcinoma are provided in Supplementary Table S2.

All five genera observed to be enriched in gastric adenocarcinoma belonged to the three predominant phyla: *Lactobacillus* (Phyla: *Firmicutes*) was reported in 25 studies (45%) in multiple biological matrices: tumour tissue (n = 16), faeces (n = 5), gastric/oesophageal fluid (n = 3), tongue coating (n = 1). *Streptococcus* (Phyla: *Firmicutes*) was identified in 23 studies (41%): tumour tissue (n = 13), faeces (n = 8), gastric/oesophageal fluid (n = 2), tongue coating (n = 1), saliva (n = 1). *Prevotella* (Phyla: *Bacteroidetes*) was present in 16 studies (29%): tumour tissue (n = 13), faeces (n = 2), saliva (n = 1). *Fusobacterium* (Phyla: *Fusobacteria*) was reported in 14 studies (25%), including tumour tissue (n = 10), faeces (n = 2), gastric/oesophageal fluid (n = 1) and urine (n = 1). *Veillonella* (Phyla: *Firmicutes*) was found in 11 studies (20%) and was reported in various biological matrices, including tumour tissue (n = 5), faeces (n = 3), gastric/oesophageal fluid (n = 2) and subgingival plaque (n = 1).

*Bacteria enriched in benign gastric conditions*: The benign group demonstrated an enrichment of 218 bacteria reported at the following levels: 12 phyla, 12 classes, 20 orders, 30 families, 93 genera, 47 species and 4 strains. No bacteria were reported in more than 10 studies.

*Bacteria enriched in healthy control group*: The healthy control group showed enrichment of 129 bacteria reported at the following levels: 13 phyla, 3 classes, 5 orders, 13 families, 62 genera and 33 species. No bacteria were reported in more than 10 studies.

Microbial diversity metrics: 51 studies (91%) assessed microbial diversity. The majority of studies employed more than one metric. Alpha diversity, a measure of the number (richness) of species within a sample and the distribution of abundances of the individual species (evenness), was reported in 50 studies (89%). Richness and evenness indices reported included: the Shannon index (n = 46); Chao1 (n = 28); OTUs (n = 21); the Simpson Index (n = 17); ACE (n = 9); the Phylogenetic Diversity tree (n = 9). All alpha diversity metrics reported different outcomes with either an increase, decrease or no change in the species richness. Overall, 13 studies demonstrated an increase in alpha diversity, 11 studies reported a decrease, 13 studies showed no difference, and 15 studies reported at least two opposing changes. Beta diversity, a measure of the species diversity between two communities or ecosystems, was measured in 42 studies (75%); principal coordinate analysis was used in 36 studies, weighted and unweighted UniFrac distance was employed in 23 studies, and Bray Curtis in 21 studies. Twenty-eight studies reported a significant difference between cancer and benign/control samples. Ten studies showed no significant difference, and two studies reported conflicting outcomes. Evaluation of diversity metrics has shown conflicting results for alpha diversity but trends towards a significant difference in beta diversity between cancer and non-cancer samples.

### 3.3. Oesophageal Adenocarcinoma

Study characteristics: Fourteen studies included the analysis of the oesophageal adenocarcinoma microbiome, including one combined SCC/OAC group (Table 2; detailed study characteristics are provided in Table S1). A total of 1223 patients were included in the analysis: 623 OAC (51%); 532 healthy controls (43%); and 68 with benign disease (6%). Half of the studies originated from Asian populations. All studies, except one, were prospective case-control studies.

Sample type: Primary sites for sample collection were from the upper gastrointestinal tract and the oral cavity. Tissue biopsy (n = 8), saliva (n = 3) and faecal (n = 2) samples were the most common, with a single study using mouthwash samples.

Method for bacterial identification: Nine of the fourteen studies identified bacteria by 16S rRNA sequencing with different targeted variable regions, V3-4 being the most common (n = 7). Other methods employed were q-PCR, RT-PCR and bacteria culture methods.

Bacteria enriched in oesophageal carcinoma: No common trend was observed at any taxonomic level for studies that evaluated bacteria related to oesophageal adenocarcinoma. A maximum of two studies shared similar bacterial identification. With relative paucity in data related to oesophageal adenocarcinoma, robust conclusions could not be drawn.

### 3.4. Oesophageal Squamous Cell Carcinoma

Study characteristics: Twenty-eight studies included the analysis of the oesophageal squamous cell carcinoma, with one combining SCC/OAC samples (Table 3; detailed study characteristics are provided in Table S1). A total of 3262 patients were included in the analysis: 2346 patients with SCC (72%); 884 healthy controls (27%); and 32 with benign disease (1%). Twenty-four of the twenty-eight studies originated from Asian populations. Three studies were retrospective in nature, evaluating publicly available datasets. The remainder of the studies were prospective case-control design.

Sample type: Primary sites for sample collection were from the upper gastrointestinal tract, tissue biopsy (n = 14), and the oral cavity, saliva (n = 8). Other bio-sample included, faecal samples (n = 4).

Method for bacterial identification: Twenty-two of the twenty-eight studies identified bacteria by 16S rRNA sequencing with different targeted variable regions, V3-4 being the most common (n = 19). Other methods employed were q-PCR, RT-PCR and bacteria culture methods.

Bacteria enriched in oesophageal squamous cell carcinoma: A total of 146 oesophageal SCC specific taxa were reported: 7 phyla, 5 classes, 10 orders, 19 families, 74 genera and 31 species. Full details of bacteria identified in association with oesophageal SCC are provided in Supplementary Table S2. Three bacterial genera were identified in six or more studies, demonstrating an enrichment of *Streptococcus* (n = 10 studies) [49,56,58], *Fusobacteria* and *Prevotella* in tissue and saliva samples [49,56,59]. No significant abundances were identified in the comparison groups.

## 4. Evaluation of Diagnostic Performance

The diagnostic performance of the microbiome was assessed by Sun et al., who reported a 97% sensitivity of saliva and subgingival plaque for gastric cancer screening [3]. No other clinically relevant applications of the microbiome were determined from studies presented herein.

## 5. Influence of Sample Origin

There was insufficient data to compare the relationship between the tumour tissue and oral microbiome of patients with oesophagogastric cancer. However, three studies showed a similarity in bacterial abundance across the orodigestive tract. Li et al. demonstrated common biomarkers, *Bosea*, *Solobacterium*, *Gemella* and *Peptostreptococcus*, in saliva and cell brush tissue in oesophageal squamous cell carcinoma patients [93]. Hao et al. identified a depletion of *Streptococcus* and an enrichment of *Veillonella*, alongside several other bacteria in the oesophageal adenocarcinoma saliva and tissue specimens [91]. Similarly, Zhang et al. supported the notion that the oral and tissue microbiome have a similar shared network [78].

## 6. Quality of Assessment

The Newcastle Ottawa Scale for case-control and cohort studies assessed the quality of reporting [104]. A total of 41 (47%) studies scored the maximum of nine stars; Ten studies 11%) scored eight stars, and 35 (40%) scored seven stars. All studies reported patient selection representative of the community population, apart from one study that enrolled hospitalized patients. Microbiome results were reported accurately in accordance with the outcome measures. Although age and gender were provided for all groups, comparability between groups, including statistical analysis of baseline characteristics, were poorly reported. No study scored less than six, suggesting overall good study design, assessment and reporting of primary outcomes.

The STORMS checklist demonstrated clear reporting of the rationale of the study (100%), detailed sample collection (91%), DNA extraction strategies (87%) and storage (61%). Few studies used positive controls (mock communities, 4%), discussed batch effects (10%) or reported contamination within DNA extraction kits/reagents, which are prevalent in low biomass samples (21%). Important microbial influencing factors such as antibiotic use (61%) and matching criteria (35%) were not reported in all studies as potential confounding factors. Although it is strongly encouraged to deposit sequence reads in an established publicly available repository such as NCBI, this was done in only 44% of studies. Full details of quality assessment scoring are provided in Supplementary Tables S3 and S4.

## 7. Discussion

This systematic review summarises the current published literature concerning the association between the microbiome and oesophagogastric cancer. The principal findings were: (i) the enrichment of select bacteria in gastric adenocarcinoma compared to benign disease and healthy controls, with distinctive bacterial profiles observed at all taxonomic levels; (ii) enrichment of the genera *Lactobacillus* and *Streptococcus* in gastric adenocarcinoma; (iii) higher abundances of *Firmicutes* in various bio-samples including tumour tissue, saliva and gastric fluid; and (iv) significant methodological heterogeneity in standards of reporting.

The microbiome has received increasing attention for its potential role in disease pathogenesis, including carcinogenesis within the gastrointestinal tract [42]. As such, intestinal bacteria may have a role in early cancer detection [3,4] and, with advancements in immunotherapy, in personalised therapeutics [7]. Early detection by profiling the salivary microbiome has been reviewed by Muszyński et al., demonstrating a distinct alteration in cancer compared to healthy controls. However, a link between the salivary and tumour microbiome is yet to be established [105]. There is also evidence suggesting the microbiome can provide prognostic information, related to a post-operative change in the microbiome linked with complications and survival [12,51,106]. Compared to the lower gastrointestinal tract, characterisation of the upper gastrointestinal microbiome remains, however, in a very preliminary stage.

Enrichment of the genera *Lactobacillus* and *Streptococcus* were common findings in 34 studies (61%) evaluating gastric adenocarcinoma. Although this review supports an important alteration in species composition, there is disagreement between species diversity. Most studies used more than one alpha diversity metric, producing conflicting results. Although it is becoming increasingly evident that microbial alteration in disease is important, assessment of the functional capabilities is deemed to provide more valuable information. The functional ecology describes the active contribution of microbes to a system but may not reflect the species composition. Species may be present; however, they can be functionally redundant.

In recent years, metagenomic analysis has transformed the understanding of cancer pathogenesis within the cancer–microbe–immune axis. PD-L1, expressed in half of all gastric cancers, are potentially responsible for suppressing killer T cells and activating regulatory T cells to promote carcinogenesis. In this setting, bacteria-enriched cancer pathways and their associated metabolites may be critical in directing tumour immunity to induce carcinogenesis [107]. Hu et al. described cancer-specific metabolites generated from pathways related to amino acid metabolism (L-arginine, L-ornithine) and the pentose phosphate pathways in gastric cancer compared to superficial gastritis [31]. Functional prediction of the microbiome using software such as PICRUSt, Tax4Fun and KEGG databases have been utilised with 16S rRNA data to explore potential cancer-mediated pathways. Barrett’s oesophagus and oesophageal adenocarcinoma are linked to the nitric oxide pathway in the context that nitrate is reduced by bacterial metabolism in the digestive tract. Downstream metabolites have been associated with oesophagogastric cancer [91]. Genomic analyses have identified the presence of bacterial genes expressing these pathways of interest. Other amino acid metabolic pathways such as cysteine and methionine metabolism have been identified in gastric cancer and shown to play a pivotal role in cancer cell growth [108]. Their end metabolites are responsible for localised inflammation, an important step in the progression to cancer. Several amino acid metabolism pathways evidenced in gastric cancer can be predictive of increased metabolism driven by the gastric microbiome.

Identification of these pathways can lead to better understanding of tumour biology and mechanisms, offering a platform for targeted therapeutics. Microbial functional analysis showed modulation of the tumour microenvironment by inflammation and cell cycle regulation. An increased functional microbial abundance of arachidonic acid, oxidative phosphorylation and the tricarboxylic acid cycle was shown in gastrointestinal cancers [109]. The pro-inflammatory effects of bacteria may be a key mechanism driving the host–microbe interactions to promote carcinogenesis through DNA replication or DNA repair [81]. Park et al. showed the enrichment of cancer pathways linked to DNA replication and mismatch repair, which are crucial to cell growth and survival [81]. Functional pathway analysis may reveal the role of the cancer microbiome in therapeutic monitoring and response.

The role of the microbiome in the pathogenesis of gastric cancer was reported by Wang et al., who identified characteristic bacterial variation that was related to disease progression from chronic gastritis to intestinal metaplasia to gastric cancer [42]. Specifically, *Actinobacteria*, *Bacteroides*, *Firmicutes* and *Fusobacteria* were enriched in premalignant and cancer states, with candidate OTUs demonstrating an area under the curve >0.9 for gastric cancer detection [42]. *Firmicutes* were also reported in higher abundances in different biological matrices including the tissue, gastric fluid and saliva of gastric cancer patients. Similarities in bacterial taxa found in different biological samples may support future non-invasive methods for characterising the microbiome in oesophagogastric cancer. Sun et al. reported that saliva and the subgingival plaque microbiome could detect gastric cancer with 97% sensitivity [3].

A limited number of studies with variable methodologies meant that fewer trends were observed in the oesophageal adenocarcinoma microbiome. Of 14 studies, only three studies identified the same seven genera, *Veillonella*, *Pseudomonas*, *Prevotella*, *Peprostreptococcus*, *Moryella*, *Leptotrichia* and *Clostridium* [58,61,91]. No bacterial changes were consistently observed in more than two studies.

A total of 41 of the 87 studies (47%) identified by this review were published since 2021, highlighting how interest in the upper gastrointestinal cancer microbiome is a relatively recent occurrence. During the emergence of this field, there has been significant heterogeneity between the methodologies used by individual studies. Though 16S rRNA gene sequencing was the most common method for defining the microbiome used in studies identified by this review, there were differences in the variable regions targeted. As different variable regions amplify different bacteria, there is a risk of taxonomic bias. Studies that also included culture methods introduce further selection bias. It is therefore recommended that a standardised methodology for sample acquisition and microbiome assessment, including DNA extraction and sequencing, should be considered. The diversity of methods used may explain the observed variation in results.

Existing checklists for the assessment of study reporting and quality do not encompass specific aspects of study design that are important to human microbiome research. Many checklists, including the Newcastle Ottawa Scale that was used to assess studies included in this review, fail to assess both patient-specific (e.g., diet, co-morbidity, medications) and analytical (e.g., sample handling, analysis and data processing) factors that have the potential to influence findings. Recently, a consortium of researchers have proposed a standardised checklist to improve reporting of human microbiome research [110]. Though the STORMS checklist has not yet been validated, it can serve as a guide to researchers within the field. The checklist comprises key features that are considered to be important in reporting high-quality and reproducible microbiome research. For this reason, the STORMS checklist was selected as the most relevant and accurate representation for quality assessment. For studies included in this review, it highlighted: (i) deficiencies in eliminating sources of contamination by using controls and assessing DNA extraction kits; (ii) the need to control for patient factors influencing the microbiome; and (iii) heterogeneity in laboratory methods used, including target variable regions. Though it is beyond the scope of the current review to establish similar guidance, important considerations for the specific analysis of the upper gastrointestinal microbiome are presented in Table 4 and are intended to serve as a guide to future investigators.

This review suffers from a number of acknowledged limitations. As previously mentioned, wide methodological variation meant that it was not possible to draw robust conclusions from the combined outcomes of selected studies. Over three quarters of included studies recruited less than eighty cancer patients, suggesting that they may have been underpowered to draw definitive conclusions. No study presented a formal sample size calculation. Furthermore, only 31 studies out of 87 (36%) used multiple comparison correction. Studies were predominantly from Far Eastern centres and, as such, have poor external validity; hence, it is uncertain how translatable results may be for patients from other global regions. Microbial profiles are influenced by many environmental factors, and geographical location encompasses many of these. With the recent increase in the incidence of oesophageal adenocarcinoma in Western countries, more studies are encouraged to define the microbial profile in the these populations [111]. A meta-analysis of the datasets of studies identified in this review was not possible due to a lack of publicly deposited datasets and associated metadata. An attempt to retrieve this information from corresponding authors was not successful. Other features of the microbiome, including the virome and the mycobiome, have not been explored, as they remain outside the scope of this work.

In conclusion, the primary limitation in microbiome research of oesophagogastric cancer is the lack of standardisation in study methodology. This heterogeneity limits the comparability of data. As a relatively new field, there is a wide scope to investigate the alterations in the human ecological system to identify cancer-related markers. The causal link between microbiome and disease and the correlative relationship between the oral cavity and the tumour tissue have not been fully explored. The results provide an insight into the importance of investigating the oesophagogastric cancer microbiome and the crucial role it plays in carcinogenesis and personalised therapeutics. Furthermore, understanding the functionality and metabolic capabilities of cancer-specific bacteria with a multi-omics approach could support improved early diagnostics and personalised treatment strategies.

## Figures and Tables

**Table 1:** Study characteristics of articles reporting on the microbiome of gastric adenocarcinoma compared to a non-cancer group.

Author	Year	Country	Specimen Type	Microbiome Assessment Method	NAT	PPI	Abx	Ref.
Sjostedt	1985	Sweden	Saliva, OG fluid	Culture	-	No	No	[20]
Dicksved	2009	Sweden	Tissue	T-RFLP	-	-	No	[21]
Seo	2014	Korea	Tissue	RNA seq database	-	-	-	[22]
Aviles-Jimenez	2014	Mexico City	Tissue	16S, microarray hybridization	-	No	No	[23]
Eun	2014	Korea	Tissue	16S V5, pyroseq	-	No	No	[24]
Hu	2015	China	Tongue coating	16S V2-4, Illumina seq	No	-	No	[25]
Wang	2016	China	Tissue	16S V1-3, pyrosequencing	-	No	No	[26]
Yu	2017	China/Mexico	Tissue	16S V3-4, MiSeq	No	-	-	[27]
Li	2017	Hong Kong	Tissue	16S V3-4, Solexa Illumina seq	-	No	No	[28]
Castano-Rodriguez	2017	Malaysia	Tissue	16S, MiSeq	No	No	No	[29]
Ferreira	2018	Portugal	Tissue	16S V5-6, NGS	-	No	No	[30]
Sun	2018	China	Saliva, SP	16S, MiSeq	-	-	No	[3]
Hu	2018	China	Gastric fluid	Shotgun, HiSeq	No	No	No	[31]
Wu	2018	China	Tongue coating	16S V4, pyrosequencing	No	No	No	[32]
Hsieh	2018	Taiwan	Tissue	16S V3-4, MiSeq	-	-	-	[33]
Coker	2018	China	Tissue	16S V4, N-W algorithm	No	No	No	[34]
Shao	2019	China	Tissue	16S V4 + miniseq	-	-	-	[35]
Gunathilake	2019	South Korea	Tissue	16S V3-4 + MiSeq	-	-	-	[36]
Liang	2019	China	Faeces	16S +MiSeq	No	No	No	[37]
Kageyama	2019	Japan	Saliva	16S V1-2 + Ion PGM Hi-Q Seq	No	-	No	[38]
Chen	2019	China	Tissue	16S V4-5 HiSeq	No	No	No	[39]
Dong	2019	China	Serum	16S V1-2 HiSeq	-	No	No	[40]
Liu	2019	China	Tissue	16S V3-4 MiSeq	No	No	No	[16]
Qi	2019	China	Faeces	16S V3-4 MiSeq	No	-	No	[41]
Wang	2020	China	Tissue	16S V4 MiSeq	No	No	No	[42]
Wang	2020	China	Tissue	16S V3-4 HiSeq	-	No	No	[43]
Spiegelhauer	2020	Denmark	Tissue	Culture + 16S V3-4 HiSeq	No	No	No	[44]
Gantuya	2020	Mongolia	Tissue	16S V3-4 + MiSeq	-	No	No	[45]
Wu	2020	China	Tissue	16S + HiSeq	No	No	No	[46]
Xu	2020	China	Tongue coating	16S V3-4 + MiSeq	No	No	No	[47]
Dang	2020	China	Tissue	16S V3-4 + MiSeq	-	-	-	[48]
Park	2021	Korea	Faeces, serum, urine	16S V3-4 + MiSeq	No	No	No	[66]
Pimenetel-Nunes	2021	Portugal	Tissue	16S V1-8	-	No	-	[67]
Yu	2021	China	Faeces	16S V3-4 + TruSeq nano	No	-	No	[63]
Yang	2021	USA	Buccal	Shotgun	-	-	No	[68]
Dai	2021	China	Tissue	16S V3-4 + Ion plus fragment	-	-	-	[69]
Li	2021	China	Tissue	16S V3-4 + MiSeq	-	No	No	[70]
Gunathilake	2021	Korea	Tissue	16S V4 + MiSeq	-	-	-	[71]
Huang	2021	China	Saliva	16S V3-4 + MiSeq	No	No	No	[72]
Oliveira	2021	North Brazil	Saliva, dental plaque	qPCR	No	-	No	[73]
Sarhadi	2021	Finland	Faeces	16S V2-4, V6-9 + Ion Chip	No	-	No	[74]
Zhang	2021	China	Tissue	16S V3-4 + MiSeq	-	-	-	[75]
Liu	2021	China	Faeces	16S V3-4 + 454 GS-FLX	-	-	-	[76]
Zhang Y	2021	China	Faeces	16S V4 + HiSeq	No	No	No	[77]
Abate	2022		FFPE tissue	MSKCC and TCGA	-	-	-	[64]
Liu	2022	China	Tissue	10 public datasets 16S	-	No	No	[65]
Zhang C	2022	China	Faeces, tissue, oral mucosal	16S V4 + Novoseq/MiSeq	No	-	No	[78]
He	2022	China	Tissue, gastric juice	16S V4 + MiSeq	-	No	No	[79]
Ding	2022	China	Faeces, gastric juice	16S V4 + NovoSeq	-	-	No	[80]
Park	2022	China	Gastric juice	16S V3-4 + MiSeq	-	-	No	[81]
Zhou	2022	China	Tissue, faeces	16S V3-4 + MiSeq	No	No	No	[82]
Sun	2022	China	Tissue	16S V3-4	No	No	No	[83]
Png	2022	Singapore	Tissue	16S V3-4 + MiSeq	-	-	-	[84]
Shu	2022	China	Saliva	16S V3-4 + Ion S5^TM^ XL	No	-	No	[85]
Zhang Z	2022	China	Faecal	16S V3-4 + MiSeq	No	No	No	[86]
Shi	2022	China	Tissue	16S V3-4 + MiSeq	No	-	No	[87]

OG, oesophagogastric. SP, subgingival plaque. B, benign. HC, healthy control. AHT, adjacent healthy tissue. 16S, 16S rRNA. N-W, Needleman–Wunsch. NAT, neoadjuvant therapy. PPI, proton pump inhibitor therapy. Abx, antibiotic therapy. Y, yes. N, no.

**Table 2:** Study characteristics of articles reporting on the microbiome of oesophageal adenocarcinoma compared to a non-cancer group.

Author	Year	Country	Specimen Type	Microbiome Assessment Method	NAT	PPI	Abx	Ref.
Yamamura	2016	Japan	Tissue	qPCR	No	-	No	[51]
Elliott	2017	UK	Tissue	16S V1-2 + MiSeq	Mixed	-	-	[52]
Peters	2017	USA	Mouthwash	16S V4 + MiSeq	-	-	-	[53]
Kageyama	2019	Japan	Saliva	16S V1-2 + Ion PGM Hi-Q Seq	No	-	No	[38]
Yuda	2020	Japan	Saliva	Culture	Mixed	-	No	[12]
Peter	2020	USA	Tissue	16S V4 + Miseq	-	Mixed	No	[57]
Li	2020	China	Tissue	16S V3-4 + Miseq	No	-	No	[58]
Zhou	2020	Australia	Tissue	16S V1-3 + Miseq	No	-	No	[60]
Lopetsu	2020	Italy	Tissue	16S V3-4 + Miseq	No	N	No	[61]
Kawasaki	2020	Japan	Subgingival plaque	RT-PCR	No	-	No	[62]
Ishaq	2021	China	Faeces	16S V3-4 + Hiseq, qPCR	-	-	No	[88]
Wang	2021	-	Tissue	TCMA database	-	-	-	[89]
Deng	2021	China	Faeces	16S V4 + Miseq	No	No	No	[90]
Hao	2022	USA	Tissue, oral mucosal	-	-	-	No	[91]

16S, 16S rRNA. NAT, neoadjuvant therapy. PPI, proton pump inhibitor therapy. Abx, antibiotic therapy. Y, yes. N, no.

**Table 3:** Study characteristics of articles reporting on the microbiome of oesophageal squamous cell carcinoma compared to a non-cancer group.

Author	Year	Country	Specimen Type	Microbiome Assessment Method	NAT	PPI	Abx	Ref.
Chen	2015	China	Saliva	16S V3-4 + pyrosequencing	No	-	No	[49]
Nasrollahzadeh	2015	Iran	Tissue	16S V3-4 + GS-FLX	-	-	-	[50]
Yamamura	2016	Japan	Tissue	qPCR	No	-	No	[51]
Shao	2019	China	Tissue	16S V4 + miniSeq	-	-	-	[35]
Kageyama	2019	Japan	Saliva	16S V1-2 + Ion PGM Hi-Q Seq	No	-	No	[38]
Wang	2019	China	Saliva	16S V3-4 + MiSeq	No	-	No	[54]
Yamamura	2019	Japan	Tissue	qPCR	Mixed	-	-	[55]
Xu	2020	China	Oral mucosal swab	16S V3-4 + Ion S5 TM XL	Yes	-	No	[56]
Yuda	2020	Japan	Saliva	Culture	Mixed	-	No	[12]
Li	2020	China	Tissue	16S V3-4 + Miseq	No	-	No	[58]
Zhao	2020	China	Saliva	16S V3-4 + Miseq	No	-	No	[59]
Kawasaki	2020	Japan	Subgingival plaque	RT-PCR	No	-	No	[62]
Li Z	2021	China	Tissue	16S V3-4 + Miseq, qPCR	-	-	-	[92]
Li Z	2021	China	Saliva, tissue	16S V4 + Ion S5^TM^ XL	-	No	No	[93]
Wei	2021	China	Saliva	16S V4 + Hiseq	-	No	No	[94]
Ishaq	2021	China	Faeces	16S V3-4 + Hiseq, qPCR	-	-	No	[88]
Jiang	2021	China	Tissue	16S V3-4	No	No	No	[95]
Wang	2021	-	Tissue	TCMA database	-	-	-	[89]
Shen	2021	China	Tissue	16S + qPCR	-	-	-	[96]
Chen	2021	China	Oral mucosal swab	16S V3-4	-	-	-	[97]
Kovaleva	2021	Russia	FFPE tissue	16S V3-4 + qPCR	-	-	-	[98]
Yang	2021	China	Tissue	16S V4	-	No	No	[99]
Deng	2021	China	Faeces	16S V4 + Miseq	No	No	No	[90]
Cheung	2022	Hong Kong	Faeces	16S V4 + Miseq	No	No	No	[100]
Wu	2022	China	Faeces	-	No	No	No	[101]
Lin	2022	China	Tissue	16S V3-4 + Hiseq	No	No	No	[102]
Shen	2022	China	Tissue	16S V1-9 + Miseq	No	-	-	[103]

FFPE, formalin-fixed paraffin-embedded. 16S, 16S rRNA. NAT, neoadjuvant therapy. PPI, proton pump inhibitor therapy. Abx, antibiotic therapy. Y, yes. N, no.

**Table 4:** Specific considerations for designing a microbiome assessment study in upper gastrointestinal disease.

Workflow		Considerations
Study design	Prospective case-control	Cross-sectional observational studies to determine microbial–disease associations Longitudinal studies: premalignant conditions to identify causative factors for diagnostic purposes, therapeutic response, prognostication
	Patient factors influencing the microbiome	Neoadjuvant treatment naive Use of proton pump inhibitors and/or histamine-2 antagonists Use of antibiotics (consider time interval between use and microbiome assessment) Immunosuppressive states Synchronous cancer Smoking status Previous gastrointestinal surgery
	Geographical location	Genetic and lifestyle factors such as diet and exercise
	Matching groups	Matched age and gender as a minimum
	Sample size calculation	
	Sub-group analysis	E.g., tumour stage, ethnicity, geographical location and its association with therapeutic response and prognostication—homogeneity will allow for a more accurate microbiome assessment.
Sampling process	Sample weight	Endoscopic tissue biopsies can be of variable size. Establish a minimum weight of tissue for adequate analysis.
	Positive control	Consider adjacent healthy tissue.
	Negative control	Consider storing an empty tube and/or storage medium at the same time as the sample.
	Replicates	(where possible)
	Minimise freeze–thaw cycles	
Laboratory techniques		This is comprehensively covered by the STORMS reporting checklist [110].

## Supplementary Materials

The following supporting information can be downloaded at: https://www.mdpi.com/article/10.3390/cancers15102668/s1, Table S1: Detailed study characteristics of articles reporting on the microbiome of gastric adenocarcinoma, oesophageal adenocarcinoma and oesophageal squamous cell carcinoma compared to a non-cancer group; Table S2: Full data extraction worksheet from identified studies in this review; Table S3: Detailed Newcastle Ottawa Scale workflow; Table S4: Detailed STORMS checklist workflow.
