# Supplementary material for: Evaluation of the Oesophagogastric Cancer-Associated Microbiome: A Systematic Review and Quality Assessment
Source: Cancers (Basel). 2023 May 9;15(10):2668. doi: 10.3390/cancers15102668 (PMC10216300; doi:10.3390/cancers15102668)
Supplement: Supplementary file 1 [file cancers-15-02668-s001.zip › Table S1_Study characteristics of article reporting on the oesophagogastri c cancer microbiome.pdf]

Table S1-1: Study characteristics of articles reporting on the microbiome of gastric adenocarcinoma compared to a non-cancer group

Author	Year	Country	Specimen type	Gastric adenocarcinoma			Control group	Control			Microbiome assessment method	MCC	No. of genera enriched (C/B/HC)	NAT	PPI	Abx
				No. of patients	Age (yrs)	Gender (male)		No. of patients	Age (yrs)	Gender (male)						
**Sjostedt**	1985	Sweden	Saliva, OG fluid	10	-	-	B, HC	30, 30	-	-	Culture	-	8/9/9	-	No	No
**Dicksved**	2009	Sweden	Tissue	10	71	8	HC	5	73	3	T-RFLP	-	-	-	-	No
**Seo**	2014	Korea	Tissue	16	60	12	AHT	-	-	-	RNA seq database	-	-	-	-	-
**Aviles-Jimenez**	2014	Mexico City	Tissue	5	71	1	B	5	44	0	16S, microarray hybridization	Yes	0/4/-	-	No	No
**Eun**	2014	Korea	Tissue	11	66	6	B	10	50	4	16S V5, pyroseq	-	2/0/-	-	No	No
**Hu**	2015	China	Tongue coating	74	57	37	HC	72	55	35	16S V2-4, Illumina seq	-	0/-/4	No	-	No
**Wang**	2016	China	Tissue	103		-	B	212	-	-	16S V1-3, pyrosequencing	-	3/0/-	-	No	No
**Yu**	2017	China/Mexico	Tissue	160	63	109	AHT	-	-	-	16S V3-4, MiSeq	-	5/-/0	No	-	-
**Li**	2017	Hong Kong	Tissue	7	53	5	B, HC	9, 8	50, 49	2, 3	16S V3-4, Solexa Illumina seq	-	9/2/8	-	No	No
**Castano-Rodriguez**	2017	Malaysia	Tissue	12	62	4	B	20	50	11	16S, MiSeq	-	24/8/-	No	No	No
**Ferreira**	2018	Portugal	Tissue	54	59	32	B	81	44	79	16S V5-6, NGS	Yes	6/5/-	-	No	No
**Sun**	2018	China	Saliva, SP	37	-	-	HC	-	-	-	16S, MiSeq	Yes	5/-/7	-	-	No
**Hu**	2018	China	Gastric fluid	6	61	5	B	5	55	4	Shotgun, HiSeq	Yes	4/4/-	No	No	No
**Wu**	2018	China	Tongue coating	57	59	40	HC	80	55	50	16S V4, pyrosequencing	Yes	6/-/23	No	No	No
**Hsieh**	2018	Taiwan	Tissue	11	69	5	B	9	32	3	16S V3-4, MiSeq	-	3/0/-	-	-	-
**Coker**	2018	China	Tissue	20	-	-	B	44	-	-	16S V4, N-W algorithm	Yes	6/2/-	No	No	No
**Shao**	2019	China	Tissue	34	62	-	AHT	-	-	-	16S V4 + miniseq	-	17/-/0	-	-	-
**Gunathilake**	2019	South Korea	Tissue	268	54	172	HC	288	52	181	16S V3-4 + MiSeq	Yes	2/-/2	-	-	-
**Liang**	2019	China	Faeces	20	52	-	HC	22	53	-	16S +MiSeq	-	3/-/1	No	No	No
**Kageyama**	2019	Japan	Saliva	10	71	6	HC	118	66	84	16S V1-2 + Ion PGM Hi-Q Seq	-	-	No	-	No
**Chen**	2019	China	Tissue	62	60	46	AHT	-	-	-	16S V4-5 HiSeq	Yes	9/-/0	No	No	No
**Dong**	2019	China	Serum	71	59	60	HC	13	-	-	16S V1-2 HiSeq	Yes	3/-/2	-	No	No
**Liu**	2019	China	Tissue	276	61	195	AHT	-	-	-	16S V3-4 MiSeq	-	7/-/0	No	No	No
**Qi**	2019	China	Faeces	116	58	96	HC	88	46	53	16S V3-4 MiSeq	-	11/-/6	No	-	No
**Wang**	2020	China	Tissue	29	57	18	B, HC	21, 30	45, 46	9, 15	16S V4 MiSeq	Yes	5/4/2	No	No	No
**Wang**	2020	China	Tissue	60	60	44	B	60	52	41	16S V3-4 HiSeq	-	16/20/-	-	No	No
**Spiegelhauer**	2020	Denmark	Tissue	12	62	7	B	22	48	4	Culture + 16S V3-4 HiSeq	Yes	1/0/-	No	No	No
**Gantuya**	2020	Mongolia	Tissue	48	-	-	B, HC	60, 20	-	-	16S V3-4 + MiSeq	-	8/0/0	-	No	No
**Wu**	2020	China	Tissue	18	63	15	B	32	62	24	16S + HiSeq	-	11/16/-	No	No	No
**Xu**	2020	China	Tongue coating	181	64	146	HC	112	64	91	16S V3-4 + MiSeq	Yes	12/-/14	No	No	No
**Dang**	2020	China	Tissue	30	-	-	B	17	-	-	16S V3-4 + MiSeq	-	4/0/-	-	-	-
**Park**	2021	Korea	Faeces, serum, urine	181	64	122	HC	272	64	159	16S V3-4 + MiSeq	Yes	19/-/7	No	No	No
**Pimenetel-Nunes**	2021	Portugal	Tissue	31	70	14	B, HC	17	53	6	16S V1-8	-	2/0/0	-	No	-
**Yu**	2021	China	Faeces	49	62	31	HC	49	-	-	16S V3-4 + TruSeq nano	-	3/-/2	No	-	No
**Yang**	2021	USA	Buccal	165	-	-	HC	323	-	-	Shotgun	-	0/-/0	-	-	No

**Dai**	2021	China	Tissue	37	66	26	AHT	-	-	-	16S V3-4 + Ion plus fragment	Yes	10/-/-	-	-	-
**Li**	2021	China	Tissue	27	63	17	B	36	46	11	16S V3-4 + MiSeq	-	36/28/-	-	No	No
**Gunathilake**	2021	Korea	Tissue	268	54	172	HC	288	52	181	16S V4 + MiSeq	Yes	0/-/0	-	-	-
**Huang**	2021	China	Saliva	99	50	64	B	101	48	51	16S V3-4 + MiSeq	Yes	2/2/-	No	No	No
**Oliveira**	2021	North Brazil	Saliva, dental plaque	192	53	119	HC	192	51	119	qPCR	-	0/-/0	No	-	No
**Sarhadi**	2021	Finland	Faeces	29	-	14	HC	13	-	3	16S V2-4, V6-9 + Ion Chip	Yes	0/-/5	No	-	No
**Zhang**	2021	China	Tissue	20	-	-	B	17	56	-	16S V3-4 + MiSeq	-	11/13/-	-	-	-
**Liu**	2021	China	Faeces	38	-	-	HC	35	-	-	16S V3-4 + 454 GS-FLX	-	13/-/2	-	-	-
**Zhang Y**	2021	China	Faeces	83	61	56	B, HC	54, 61	55, 54	27, 33	16S V4 + HiSeq	-	28/2/0	No	No	No
**Abate**	2022		FFPE tissue	520	-	342	AHT	-	-	-	MSKCC and TCGA	Yes	16/-/-	-	-	-
**Liu**	2022	China	Tissue	438	61	200	B, HC	318, 109	50, 46	151, 56	10 public datasets 16S	Yes	22/0/0	-	No	No
**Zhang C**	2022	China	Faeces, tissue, oral mucosal	76	64	51	HC	70	61	36	16S V4 + Novoseq/MiSeq	Yes	5/-/3	No	-	No
**He**	2022	China	Tissue, gastric juice	64	-	-	B	61	-	-	16S V4 + MiSeq	-	15/12/-	-	No	No
**Ding**	2022	China	Faeces, gastric juice	40	-	30	HC	10	-	-	16S V4 + NovoSeq	-	7/-/3	-	-	No
**Park**	2022	China	Gastric juice	56	60	39	B	16	60	6	16S V3-4 + MiSeq	Yes	3/4/-	-	-	No
**Zhou**	2022	China	Tissue, faeces	25	62	14	B	25	59	15	16S V3-4 + MiSeq	-	2/0/-	No	No	No
**Sun**	2022	China	Tissue	13	72	5	B	65	-	32	16S V3-4	Yes	3/0/-	No	No	No
**Png**	2022	Singapore	Tissue	4	-	-	B	17	-	-	16S V3-4 + MiSeq	Yes	4/1/-	-	-	-
**Shu**	2022	China	Saliva	33	-	-	HC	34	-	-	16S V3-4 + Ion S5^{TM} XL	-	2/-/3	No	-	No
**Zhang Z**	2022	China	Faecal	20	61	15	HC	30	59	15	16S V3-4 + MiSeq	-	2/-/1	No	No	No
**Shi**	2022	China	Tissue	10	-	10	AHT	-	-	-	16S V3-4 + MiSeq	-	7/-/-	No	-	No

OG, oesophagogastric. SP, Subgingival plaque. B, benign, HC, healthy control. AHT, adjacent healthy tissue. 16S, 16S rRNA. N-W, Needleman-Wunsch. MCC, multiple comparisons correction. NAT, neoadjuvant therapy. PPI, proton pump inhibitor therapy. Abx, Antibiotic therapy. Y, yes. N, no.

Table S1-2: Study characteristics of articles reporting on the microbiome of oesophageal adenocarcinoma compared to a non-cancer group

Author	Year	Country	Specimen type	Histological subtype	Oesophageal carcinoma			Control group	Control			Microbiome assessment method	MCC	No. of genera enriched (C/B/HC)	NAT	PPI	Abx
					No. of patients	Age (yrs)	Gender (male)		No. of patients	Age (yrs)	Gender (male)						
**Yamamura**	2016	Japan	Tissue	Adeno	300	66	287	HC	-	-	-	qPCR	-	0/-/7	No	-	No
**Elliott**	2017	UK	Tissue	Adeno	19	70	15	HC	20	57	7	16S V1-2 + MiSeq	Yes	0/-/0	Mixed	-	-
**Peters**	2017	USA	Mouthwash	Adeno	81	68	75	HC	210	68	168	16S V4 + MiSeq	-	1/-/4	-	-	-
**Kageyama**	2019	Japan	Saliva	Unspecified	12	68	8	HC	118	66	84	16S V1-2 + Ion PGM Hi-Q Seq	-	0/-/0	No	-	No
**Yuda**	2020	Japan	Saliva	Adeno	78	64	572	-	-	-	-	Culture	-	0/-/-	Mixed	-	No
**Peter**	2020	USA	Tissue	Adeno	10	65	9	HC	10	52	6	16S V4 + Miseq	Yes	0/-/3	-	Mixed	No
**Li**	2020	China	Tissue	Adeno	11	61	8	HC	16	59	10	16S V3-4 + Miseq	-	11/-/8	No	-	No
**Zhou**	2020	Australia	Tissue	Adeno	6	67	6	B, HC	31, 16	-, 52	-, 2	16S V1-3 + Miseq	-	3/4/3	No	-	No
**Lopetsu**	2020	Italy	Tissue	Adeno	6	56	4	HC	10	52	6	16S V3-4 + Miseq	-	5/-/3	No	N	No
**Kawasaki**	2020	Japan	SP	Adeno	3	66	47	HC	62	70	50	RT-PCR	-	0/-/0	No	-	No
**Ishaq**	2021	China	Faeces	Adeno	4	-	-	HC	20	-	6	16S V3-4 + Hiseq, qPCR	-	2/-/2	-	-	No
**Wang**	2021	-	Tissue	Adeno	20	73	15	AHT	-	-	-	TCMA database	Yes	0/-/-	-	-	-
**Deng**	2021	China	Faeces	Adeno	2	-	-	HC	23	64	4	16S V4 + Miseq	-	11/-/10	No	No	No
**Hao**	2022	USA	Tissue, OM swab	Adeno	19	60	18	B, HC	27	56	17	-	Yes	31/0/2	-	-	No

OM, oral mucosa. SP, Subgingival plaque. SCC, squamous cell carcinoma. Adeno, adenocarcinoma. B, benign, HC, healthy control. 16S, 16S rRNA. MCC, multiple comparisons correction. NAT, neoadjuvant therapy. PPI, proton pump inhibitor therapy. Abx, Antibiotic therapy. Y, yes. N, no.

Table S1-3: Study characteristics of articles reporting on the microbiome of oesophageal squamous cell carcinoma compared to a non-cancer group

Author	Year	Country	Specime n type	Histological subtype	Oesophageal carcinoma			Control group	Control			Microbiome assessment method	MCC	No. of genera enriched (C/B/HC)	NAT	PPI	Abx
					No. of patients	Age (yrs)	Gender (male)		No. of patients	Age (yrs)	Gender (male)						
**Chen**	2015	China	Saliva	SCC	87	65	59	HC	85	66	62	16S V3-4 + pyrosequencing	-	7/-/26	No	-	No
**Nasrollahzadeh**	2015	Iran	Tissue	SCC	37	65	18	B, HC	17, 37	64, 62	16, 18	16S V3-4 + GS-FLX	Yes	0/0/0	-	-	-
**Yamamura**	2016	Japan	Tissue	SCC	12	66	287	HC	-	-	-	qPCR	-	0/-/7	No	-	No
**Shao**	2019	China	Tissue	SCC	61	62	-	-				16S V4 + miniSeq	-	2/-/-	-	-	-
**Kageyama**	2019	Japan	Saliva	Unspecified	12	68	8	HC	118	66	84	16S V1-2 + Ion PGM Hi-Q Seq	-	0/-/0	No	-	No
**Wang**	2019	China	Saliva	SCC	20	66	14	HC	21	65	12	16S V3-4 + MiSeq	-	3/-/2	No	-	No
**Yamamura**	2019	Japan	Tissue	SCC	551	66	459	-	-	-	-	qPCR	-	0/-/-	Mixed	-	-
**Xu**	2020	China	OM swab	SCC	31	-	24	-	-	-	-	16S V3-4 + Ion S5 TM XL	-	3/-/-	Yes	-	No
**Yuda**	2020	Japan	Saliva	SCC	567	64	572	-	-	-	-	Culture	-	0/-/-	Mixed	-	No
**Li**	2020	China	Tissue	SCC	17	61	12	HC	16	59	10	16S V3-4 + Miseq	-	11/-/8	No	-	No
**Zhao**	2020	China	Saliva	SCC	39	60	23	HC	51	49	23	16S V3-4 + Miseq	Yes	24/-/11	No	-	No
**Kawasaki**	2020	Japan	SP	SCC	58	66	47	HC	62	70	50	RT-PCR	-	0/-/0	No	-	No
**Li Z**	2021	China	Tissue	SCC	111	63	64	-	-	-	-	16S V3-4 + Miseq, qPCR	-	3/-/-	-	-	-
**Li Z**	2021	China	Saliva, tissue	SCC	70	64	46	HC	82	59	48	16S V4 + Ion S5^{TM} XL	Yes	22/-/-	-	No	No
**Wei**	2021	China	Saliva	SCC	90	62	71	HC	50	44	27	16S V4 + Hiseq	-	4/-/-	-	No	No
**Ishaq**	2021	China	Faeces	SCC	11	-	-	HC	20	-	6	16S V3-4 + Hiseq, qPCR	-	0/-/2	-	-	No
**Jiang**	2021	China	Tissue	SCC	32	56	20	B, HC	15, 21	56, 48	9, 13	16S V3-4	-	5/3/8	No	No	No
**Wang**	2021	-	Tissue	SCC	40	60	36	-	-	-	-	TCMA database	Yes	0/-/-	-	-	-
**Shen**	2021	China	Tissue	SCC	21	66	18	HC	17	64	15	16S + qPCR	-	3/-/0	-	-	-
**Chen**	2021	China	OM swab	SCC	156	-	-	-	-	-	-	16S V3-4	-	0/-/-	-	-	-
**Kovaleva**	2021	Russia	FFPE tissue	SCC	48	61	36	-	-	-	-	16S V3-4 + qPCR	-	1/-/-	-	-	-
**Yang**	2021	China	Tissue	SCC	38	62	27	HC	15	48	9	16S V4	Yes	15/-/8	-	No	No
**Deng**	2021	China	Faeces	SCC	18	66	4	HC	23	64	4	16S V4 + Miseq	-	0/-/9	No	No	No
**Cheung**	2022	Hong Kong	Faeces	SCC	15	69	15	HC	16	60	16	16S V4 + Miseq	-	6/-/4	No	No	No
**Wu**	2022	China	Faeces	SCC	40	46	26	HC	40	46	25	-	-	1/-/2	No	No	No
**Lin**	2022	China	Tissue	SCC	120	61	89	-	-	-	-	16S V3-4 + Hiseq	Yes	2/-/-	No	No	No
**Shen**	2022	China	Tissue	SCC	19	-	12	-	-	-	-	16S V1-9 + Miseq	-	5/-/-	No	-	-

OM, oral mucosa. SP, Subgingival plaque. FFPE, Formalin fixed paraffin embedded. SCC, squamous cell carcinoma. Adeno, adenocarcinoma. B, benign, HC, healthy control. 16S, 16S rRNA. MCC, multiple comparisons correction. NAT, neoadjuvant therapy. PPI, proton pump inhibitor therapy. Abx, Antibiotic therapy. Y, yes. N, no.
